# Experience and outcomes of kidney transplantation in a Colombian referral center

**DOI:** 10.3389/fneph.2026.1817080

**Published:** 2026-05-26

**Authors:** Maricel Licht-Ardila, Diana Carolina Velasquez-Romero, Alexandra Hurtado-Ortiz, Alejandra Mendoza-Monsalve, Katherine del Consuelo Camargo-Hernández, Claudia Constanza Velasco-Martínez, Adalberto Peña Wilches, Paola Andrea Parra, Anderson Bermon, Juan Sebastian Gelvez-Rueda, Edgar Fabián Manrique-Hernández, Wilmer Roberto Rivero-Rodrigez

**Affiliations:** 1 Department of Epidemiology, Hospital Internacional de Colombia HIC- Fundación Cardiovascular de Colombia FCV- Fundación Universitaria FCV, Piedecuesta, Santander, Colombia; 2 Universidad Autónoma de Bucaramanga - UNAB, Facultad de Ciencias de la Salud, Bucaramanga, Santander, Colombia; 3 Kidney Transplant Unit, Hospital Internacional de Colombia HIC- Fundación Cardiovascular de Colombia FCV- Fundación Universitaria FCV, Piedecuesta, Santander, Colombia

**Keywords:** graft rejection, graft survival, kidney transplantation, postoperative complications, survival rate

## Abstract

**Background:**

Kidney transplantation remains the most effective therapy for End-Stage Renal Disease. While global outcomes have improved, data from Latin America are limited.

**Objective:**

to describe the institutional experience of a national kidney transplant program in Colombia and to analyze patient and graft survival, acute rejection, and graft loss over time.

**Methods:**

The objective of this research was to describe the institutional experience of a national kidney transplant program in Colombia and to analyze patient, graft, and rejection-free survival across nearly two decades. A retrospective cohort study included all adults who underwent kidney transplantation at a national referral center in Colombia between April 2006 and 5 October 2025. Outcomes were overall, graft, and rejection-free survival, analyzed with Kaplan–Meier and competing-risk models, and compared across three transplant eras (2006–2011, 2012–2018, 2019–2025).

**Results:**

A total of 422 kidney transplant recipients were included (38.4% female, 61.6% male). Renal vascular disease was the leading cause of kidney failure (38.5%). Kaplan–Meier analyses showed no sex differences; 1-year patient survival was 92.1% (95% CI: 87.8–94.9) in males and 96.7% (95% CI: 92.3–98.6) in females (p = 0.082). Era-stratified analyses demonstrated significant improvement in 1-year graft survival (log-rank p = 0.015) and a trend toward higher rejection-free survival, reaching 93.8% (95% CI: 88.4–96.7) in 2019–2025 (p = 0.062). In competing-risk analysis, death was more frequent than graft failure; at 15 years, cumulative incidence was 42.2% for death versus 17.8% for graft failure.

**Conclusion:**

Over 19 years, this program achieved survival outcomes comparable to international standards, with progressive improvement and sex-equitable results. Late mortality, rather than graft failure, has emerged as the main long-term challenge.

## 1 Introduction

Kidney transplantation is widely recognized as the most effective renal replacement therapy for patients with End-Stage Renal Disease (ESRD). Compared with long-term dialysis, it provides substantial advantages in survival, quality of life, and cost-effectiveness (1, 2). Over recent decades, significant advances in donor-recipient matching, surgical techniques, and immunosuppressive regimens have led to remarkable improvements in early outcomes, with reduced acute rejection rates and enhanced short-term graft function (2–4).

Rejection, however, remains a central determinant of transplant success. Although the incidence of acute rejection has decreased, these episodes are still associated with inferior long-term outcomes and higher risk of graft dysfunction (5). In Spain, overall mortality among patients receiving renal replacement therapy between 2013 and 2022 ranged from 7.4% to 8.4%. In 2022, the main causes of death were infections (27.8%), followed by heart or myocardial failure (17.2%), unknown causes (11.7%), tumors (9.6%), and cardiac arrest (8.6%) (6). In the United States, according to the United States Renal Data System – National Institute of Diabetes and Digestive and Kidney Diseases (USRDS–NIDDK, 2024) report, among individuals aged 75 years and older, 41% received a kidney transplant and 29% died within five years. The analysis also revealed differences by blood group, showing a cumulative incidence of kidney transplantation of 72.4% in group AB compared with 43.6% in group O, whereas mortality was lower among group AB (12.3%) than group O (19%) recipients (7).

Antibody-mediated rejection has gained particular attention as a leading cause of late graft loss, often resistant to treatment and requiring sophisticated diagnostic approaches (8, 9). Beyond immunological injury, other complications such as opportunistic infections, BK virus nephropathy, and drug toxicity also play a critical role in compromising graft function and patient survival, reflecting the delicate balance between adequate immunosuppression and treatment-related risks (10).

Recipient-related factors further influence outcomes. Comorbidities such as obesity, diabetes, and cardiovascular disease increase post-transplant morbidity and mortality, often becoming major competing risks despite a functioning graft (11). These multidimensional challenges underscore the importance of continuous monitoring and individualized care strategies to improve both patient and graft survival.

In Latin America, and particularly in Colombia, kidney transplantation programs face additional challenges linked to organ donation rates, access to care, and socioeconomic disparities. Regional variations in health systems and the burden of infectious diseases may influence transplant outcomes differently than in high-income countries (12, 13). Nonetheless, several centers in the region have reported outcomes comparable to international standards, emphasizing the value of institutional experience and the need for locally generated data (13).In this context, this study aims to describe the institutional experience of a national kidney transplant program in Colombia and to analyze patient survival, graft survival, rejection, and graft loss over time. Importantly, this study provides one of the longest follow-up institutional cohorts reported in Colombia, evaluates temporal trends across nearly two decades of transplant practice, and incorporates competing-risk modeling to account for death as a key competing event. These features distinguish our findings from prior Colombian and regional registry-based reports, which are often limited to shorter follow-up periods or descriptive analyses without longitudinal or competing-risk approaches, thereby providing more robust long-term and context-specific evidence from a middle-income Latin American setting.

## 2 Methods

A retrospective cohort study was conducted at a national referral center for kidney transplantation in Colombia, which follows standardized clinical protocols and a comprehensive, multidisciplinary model of care. The renal transplant program operates under rigorous institutional guidelines to ensure high-quality standards, patient safety, and continuity of care across all phases of the transplantation process, from preoperative evaluation and surgery to postoperative follow-up and long-term management.

The multidisciplinary transplant team comprises urologic transplant surgeons, nephrologists, anesthesiologists, infectious disease specialists, immunologists, pharmacists, pathologists, nutritionists, social workers, psychologists, and psychiatrists. Throughout the study period, kidney transplant procedures were performed within the same institutional transplant program, with participation of different surgeons over time as part of the transplant surgery team. Nephrological management followed institutional protocols that were periodically updated according to international guidelines, while preserving the same overall management framework and multidisciplinary approach throughout the study period.

Additional subspecialists, including cardiologists, radiologists, gastroenterologists, gynecologists, oncologists, and dentists, are involved as clinically indicated. The program is supported by a transplant nurse coordinator, bedside nurses with specialized training in transplantation to ensure comprehensive evaluation, education, and follow-up. All adult recipients (≥18 years) who underwent kidney transplantation between April 2006 and October 2025 were included, encompassing both deceased-donor and living-donor procedures.

### 2.1 Eligibility criteria

For the purposes of this study, all adult patients (≥18 years) who underwent kidney transplantation at our institution between April 2006 and October 2025 were included. Both deceased-donor and living-donor transplants were considered. No additional exclusion criteria were applied, and all consecutive transplant recipients during the study period were included in the analysis. The following criteria describe the institutional selection process for kidney transplantation and are provided to contextualize the study population. Eligibility for transplantation was determined through multidisciplinary evaluation. Candidates included adults with advanced chronic kidney disease (CKD) stages G4–G5 with an estimated glomerular filtration rate (eGFR) <30 mL/min/1.73 m² and expected progression; CKD G4 with eGFR <20 mL/min/1.73 m² without likelihood of recovery; or established end-stage kidney disease (ESKD) on dialysis who met the following: (1) acceptable surgical and anesthetic risk with optimized comorbidity control; (2) adequate cardiopulmonary function without uncontrolled symptomatic heart or lung disease; (3) absence of active infection, confirmed through comprehensive screening for tuberculosis (TB), human immunodeficiency virus (HIV), hepatitis B virus (HBV), hepatitis C virus (HCV), cytomegalovirus (CMV), Epstein–Barr virus (EBV), syphilis, and Chagas disease when indicated, with up-to-date vaccination; (4) absence of active malignancy, with prior cancers treated with curative intent and an appropriate disease-free interval; (5) favorable surgical feasibility with acceptable vascular and abdominal anatomy; (6) completed immunologic assessment including ABO blood group (ABO) compatibility, molecular human leukocyte antigen (HLA) typing, anti-HLA antibodies, sensitizing events, and crossmatch strategy; and (7) behavioral and social readiness, including adherence capacity, tobacco cessation when applicable, and adequate psychosocial support. Candidates meeting these criteria were activated on the national waiting list or scheduled for living-donor transplantation according to allocation policies and clinical priority.

Transplantation was contraindicated when procedural risk outweighed potential benefit relative to remaining on dialysis. Absolute contraindications included severe cardiovascular disease (advanced heart failure, non-revascularizable coronary artery disease, active cerebrovascular disease, or severe peripheral vascular disease), decompensated chronic obstructive pulmonary disease (COPD), severe pulmonary hypertension, decompensated chronic liver disease, uncontrolled active infection (including TB, viral hepatitis, or untreated HIV), active malignancy except for selected low-grade tumors in remission, absence of adequate social support to ensure adherence, severe untreated psychiatric illness, or technical impossibility of graft implantation.

Relative contraindications, requiring individualized optimization, included age >70 years with frailty or multiple comorbidities; prior malignancy requiring an adequate disease-free interval; severe but stable cardiovascular or peripheral vascular disease; refractory heart failure (considering combined heart–kidney transplantation); severe COPD; active peptic ulcer disease; coagulation disorders; prior graft loss due to recurrent focal segmental glomerulosclerosis or hemolytic uremic syndrome; cognitive impairment with limited support; documented nonadherence; psychiatric disorders requiring stabilization; HIV infection pending virologic control and immune recovery; active alcohol use without ≥6 months of abstinence and rehabilitation; primary renal diseases with high recurrence risk; and contraindications to essential immunosuppressants. Patients with modifiable conditions were re-evaluated after adequate optimization.

### 2.2 Data source and variables

The Transplant Service maintains a REDCap database (project PID 1510) (14, 15), hosted on the institutional server. This database is populated by trained clinical staff who systematically extract information from patients’ electronic medical records within the institutional health information system. The epidemiology team conducts regular audits to ensure data quality, completeness, and validity.

For analytical purposes, variables were grouped into predefined domains:

Sociodemographic variables: These included sex, age, educational level, marital status, area of residence, and body mass index (BMI). BMI was categorized as underweight (<18.5 kg/m²), normal weight (18.5–24.9 kg/m²), overweight (25.0–29.9 kg/m²), and obesity (≥30.0 kg/m²).

Clinical variables: Baseline clinical characteristics included the cause of end-stage renal disease (ESRD) and comorbidities such as hypertension, diabetes mellitus, obesity, coronary artery disease, peripheral vascular disease, cerebrovascular disease, cancer, peptic ulcer disease, heart failure, autoimmune disease, benign prostatic hyperplasia, human immunodeficiency virus (HIV) infection, hepatitis B virus (HBV), hepatitis C virus (HCV), alcoholism, and prior smoking.

Pre-transplant variables: Pre-transplant characteristics included history of blood transfusions, dialysis requirement, and dialysis modality.

Donor-related variables: These included donor type (living or deceased), donor age, sex, and serum creatinine levels.

Transplant-related variables: These comprised year of transplantation, type of transplant, cold ischemia time, warm ischemia time, need for Delayed graft function (DGF), defined as the need for dialysis within the first 7 days after transplantation); length of hospital stay, immunosuppressive regimen, graft function, and recipient serum creatinine at hospital discharge.

Outcomes: Primary outcomes included patient survival, graft survival, graft loss, and biopsy-proven acute rejection. Graft loss was defined as irreversible graft failure requiring dialysis or retransplantation. For temporal comparisons, the year of transplantation was categorized into three eras (2006–2011, 2012–2018, and 2019–2025), enabling the evaluation of longitudinal trends in graft survival and rejection-free survival in relation to advances in clinical practice and immunosuppressive strategies.

### 2.3 Immunosuppressive management followed institutional clinical practice

The standard maintenance regimen included mycophenolate mofetil 1 g orally every 12 hours or mycophenolate sodium 720 mg every 12 hours, initiated on day 0. Prednisone was started at 30 mg orally once daily on day 3 and tapered to 20 mg/day on postoperative days 7–29, 15 mg/day on days 30–60, 10 mg/day on days 61–90, and 5 mg/day after day 90, with faster tapering considered in selected patients. Extended-release tacrolimus was initiated at 0.1 mg/kg/day orally every 24 hours, on day 0 if the cold ischemia time was <24 hours or on day 1 otherwise, and subsequently adjusted based on trough-level monitoring. Tacrolimus levels were routinely measured approximately 96 hours after initiation, immediately before the scheduled morning dose. Immunosuppressive therapy followed institutional clinical practice and generally included tacrolimus, mycophenolate, and corticosteroids. However, specific tacrolimus target trough ranges were not systematically captured in the registry and were therefore not included in the analysis.

### 2.4 Surgical procedure

For deceased-donor organ procurement, the surgical team includes a transplant surgeon, operative coordinator, surgical assistant, anesthesiologist, scrub nurse, and circulating nurse. The team is responsible for donor mobilization, organ recovery, and preservation under standardized protocols. Organs are flushed with a cold preservation solution (Custodiol or Belzer), packed in triple sterile bags, maintained at 4 °C, and transported in ice-cooled containers. Logistics involve dedicated rescue kits, preservation solutions, and dual coolers (for organ and perfusion solutions). Proper labeling and chain of custody are maintained to ensure identification and safe delivery to the operating room.

### 2.5 Follow-up

Post-transplant follow-up is conducted through the outpatient transplant clinic and telephone contact. The visit frequency follows a standardized minimum schedule: weekly during the first month after discharge; every two weeks during months 2 and 3; monthly up to month 12; and every 1 to 3 months thereafter, depending on clinical stability. Surveillance includes clinical assessment, laboratory monitoring, therapeutic drug-level evaluation, and protocol or indication biopsies when required. When follow-up is discontinued or patients are unreachable, telephone contact is attempted with them or their families. If survival information cannot be obtained through this means, verification is performed using national health information databases to ensure the completeness and accuracy of the data.

### 2.6 Definitions and adjudication

Rejection episodes were adjudicated by transplant pathologists according to the contemporaneous Banff classification (16). Graft loss was defined as irreversible loss of graft function requiring dialysis or retransplantation (17).

### 2.7 Statistical analysis

Categorical variables were summarized as counts and percentages; continuous variables as median (IQR), as appropriate. Normality of continuous variables was first assessed using the Shapiro–Wilk test. Group comparisons employed χ² or Fisher’s exact test for categorical data and Mann–Whitney U test for continuous data. Survival analyses for patient survival, graft survival, and rejection-free survival were performed using Kaplan–Meier estimators, with comparisons between sex and transplant eras (2006-2011; 2012-2018; 2019-2025), conducted through log-rank tests. Time-to-event outcomes were calculated from the date of transplantation to the occurrence of each event of interest: death, graft loss, or first biopsy-proven acute rejection or censored at the last follow-up visit.

Competing-risk analyses (Fine–Gray) were considered for graft loss with death as a competing event. Missing data <10% were handled with complete-case analysis; when >10%, multiple imputation by chained equations (MICE) was applied. Imputed variables included history of blood transfusions and DGF. Statistical significance was set at p<0.05. Analyses were performed using Stata 16 (StataCorp, College Station, TX, USA) and R software.

### 2.8 Ethical considerations

The protocol received approval from the Institutional Research Ethics Committee of (2024-016-17). Procedures complied with national regulations and the Declaration of Helsinki. Data were anonymized prior to analysis.

## 3 Results

From 2006 to 2025, a total of 422 kidney transplant recipients were included, of whom 38.39% were female and 61.61% male. Male recipients were significantly older than females (median 53 vs. 44 years; p < 0.001). Hypertension was the most frequent comorbidity (66.35%), while diabetes mellitus (16.92% vs. 9.26%, p = 0.027) and smoking history was more prevalent among men (p < 0.001). Regarding the ESRD, renal vascular disease, including hypertensive nephroangiosclerosis, was the leading cause overall (38.48%), with a higher prevalence in women (41.61% vs. 36.54%; p = 0.002). Conversely, diabetes was more frequent among men (15.77% vs. 6.21%), whereas systemic lupus erythematosus predominated in women (9.94% vs. 1.54%). Peripheral vascular disease, stroke, peptic ulcer disease, autoimmune disease, or hepatitis B infection were reported in either men or women (**Table 1**).

**Table 1:** Transplant-related and donor characteristics by sex.

Variable/Category	Male 260 (61.61%)	Female 162 (38.38%)	Total (n=422)	P-value
Demographic characteristics				
Age (years)*	53 (41-66)	44 (38-56)	49.5 (39-64)	<0.001
Educational level				
Secondary	83 (22.30)	53 (14.2)	136 (36.6)	0.697
Primary	80 (21.50)	41 (11.0)	121 (32.5)	
Technical	30 (8.10)	18 (4.8)	48 (12.9)	
Professional	25 (6.70)	20 (5.4)	45 (12.1)	
Postgraduate	4 (1.10)	3 (0.8)	7 (1.9)	
No Information	3 (0.80)	5 (1.30)	8 (2.2)	
None	4 (1.10)	3 (0.80)	7 (1.9)	
Marital status				
Single	73 (28.40)	58 (37.40)	131 (31.8)	0.326
Separated	13 (5.10)	7 (4.50)	20 (4.9)	
Married	108 (42.00)	55 (35.50)	163 (39.6)	
Common-law union	53 (20.60)	30 (19.40)	83 (20.1)	
Widowed	4 (1.60)	4 (2.60)	8 (1.9)	
Not declared	6 (2.30)	1 (0.60)	7 (1.7)	
Area of residence				
Urban	231 (56.1)	141 (34.2)	372 (90.3)	0.719
Rural	26 (6.3)	14 (3.4)	40 (9.7)	
BMI				
Underweight	12 (4.62)	19 (11.73)	31 (7.35)	0.059
Normal weight	103 (39.62)	60 (37.04)	163 (38.63)	
Overweight	57 (21.92)	33 (20.37)	90 (21.33)	
Obesity	88 (33.85)	50 (30.86)	138 (32.70)	
Causes of ESRD				
Renal vascular disease (includes nephroangiosclerosis due to hypertension)	95 (36.54)	67 (41.61)	162 (38.48)	0.002
Unknown	57 (21.92)	30 (18.63)	87 (20.67)	
Diabetes	41 (15.77)	10 (6.21)	51 (12.11)	
Other glomerulonephritis	20 (7.69)	9 (5.59)	29 (6.89)	
Polycystic kidney disease	9 (3.46)	11 (6.83)	20 (4.75)	
Systemic lupus erythematosus	4 (1.54)	16 (9.94)	20 (4.75)	
Focal segmental glomerulosclerosis	6 (2.31)	6 (3.73)	12 (2.85)	
IgA nephropathy	5 (1.92)	2 (1.24)	7 (1.66)	
Familial or genetic glomerulopathy (includes Alport)	6 (2.31)	1 (0.62)	7 (1.66)	
Others	17(6.54)	6 (3.70)	27 (6.40)	
Clinical history				
Hypertension	175 (67.35)	105 (64.81)	280 (66.35)	0.598
Diabetes	44 (16.92)	15 (9.26)	59 (13.98)	0.027
Coronary artery disease	3 (1.15)	0 (0.00)	3 (0.71)	0.170
Cancer	2 (0.77)	3 (1.85)	5 (1.18)	0.318
Heart failure	5 (1.92)	1 (0.62)	6 (1.42)	0.270
Benign prostatic hyperplasia	4 (1.54)	0 (0.00)	4 (0.95)	0.113
HIV infection	1 (0.38)	0 (0.00)	1 (0.24)	0.429
Hepatitis C	1 (0.38)	0 (0.00)	1 (0.24)	0.429
Alcoholism	4 (1.54)	0 (0.00)	4 (0.95)	0.113
Prior smoking	33 (12.69)	3 (1.85)	36 (8.53)	<0.000
Pretransplant factors				
History of blood transfusions	182 (91.00)	103 (83.70)	285 (91.00)	0.026
Dialysis requirement	190 (96.45)	113 (96.58)	303 (96.50)	0.950
Dialysis modality+				
Hemodialysis	119 (45.77)	66 (40.74)	185 (43.84)	0.311
Peritoneal dialysis	84 (32.31)	58 (35.80)	142 (33.65)	0.460
Donor-related variables				
Donor type				
Living donor	4 (1.54)	11 (6.79)	15 (3.55)	0.005
Deceased donor	256 (98.46)	151 (93.21)	407 (96.45)	
Donor age (years)*	40 (25-52)	33 (24-47)	37 (25-50)	0.069
Donor sex				
Male	135 (69.95)	75 (71.43)	210 (70.47)	0.789
Female	58 (30.05)	30 (28.57)	88 (29.53)	
Donor creatinine level (mg/dL)*	0.74 (0.6-0.9)	0.79 (0.6-0.99)	0.76 (0.6-0.92)	0.176
Transplant-related variables				
Year of transplantation				
2006-2011	56 (21.54)	35 (21.60)	91 (21.56)	0.100
2012-2018	83 (31.92)	67 (41.36)	150 (35.55)	
2019-2025	121 (46.54)	60 (37.04)	181 (42.89)	
Type of transplant				
Heterotopic	191 (95.50)	116 (94.31)	307 (95.05)	0.912
Kidney–Heart	2 (1.00)	1 (0.81)	3 (0.93)	
Kidney–Pancreas	4 (2.00)	4 (3.25)	8 (2.48)	
Retransplant	3 (1.50)	2 (1.63)	5 (1.55)	
Cold ischemia time (minutes)*	864 (631-1040)	807 (610-1075)	851.5 (616-1060)	0.580
Warm ischemia time (minutes)*	32 (23-45)	30 (22-40)	30 (22-45)	0.379
DGF (yes/no)	29 (14.5)	7 (5.7)	36 (13.00)	0.026
The recipient’s serum creatinine level at hospital discharge (mg/dL)*	1.59 (1.28-2.25)	1.07 (0.815-1.50)	1.42 (1.04-2.03)	<0.000
Length of hospital stay (days)*	9 (7-14)	8 (7-14)	9 (7-14)	0.279
Immunosuppressive regimen				
Mycophenolate	198 (76.15)	121 (74.69)	319 (75.59)	0.734
Tacrolimus	196 (75.38)	120 (74.07)	316 (74.88)	0.763
Prednisolone	196 (75.38)	120 (74.07)	316 (74.88)	0.763
Everolimus	3 (1.15)	1 (0.62)	4 (0.95)	0.580
Azathioprine	1 (0.38)	0 (0.00)	1 (0.24)	0.429
Sirolimus	1 (0.38)	0 (0.00)	1 (0.24)	0.429
Cyclosporine	1 (0.38)	1 (0.62)	2 (0.47)	0.735
Belatacept	0 (0.00)	1 (0.62)	1 (0.24)	0.205

*Median (Intercuartile range:IQR). +A patient could have one or both dialysis modalities. Age: years; Body Mass Index: BMI; Donor age: years; Donor creatinine level: milligrams per deciliter (mg/dL); Cold ischemia time: minutes; Warm ischemia time: minutes; Length of hospital stay: days; Delayed graft function (defined as the need for dialysis within the first 7 days after transplantation), ESRD: End state renal disease.

Pretransplant factors also differed by sex: a history of blood transfusions was more frequent among men (91.00% vs. 83.70%; p = 0.026), and the recipient’s serum creatinine level at hospital discharge were higher in male recipients (1.59 mg/dL vs. 1.07 mg/dL; p < 0.001). Most transplants involved deceased donors (96.45%). Of the patients who received a living donor transplant, most were female recipients (81% vs. 19%; p = 0.005). No significant differences were observed in ischemia times, hospital stay, or use of immunosuppressive agents, which were predominantly mycophenolate, tacrolimus, and prednisolone (all > 74%) (**Table 1**).

**Figure 1:**
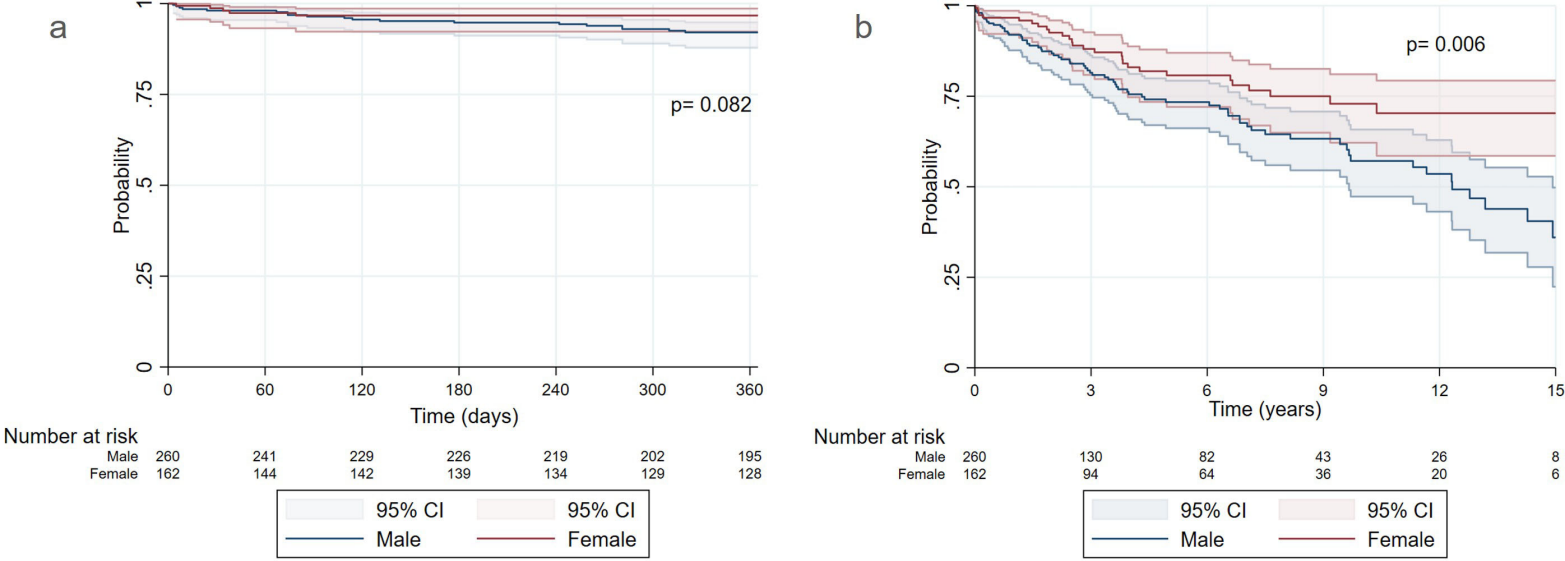
Kaplan–Meier survival curves by sex in kidney transplant recipients. **(a)** Patient survival during the first year post-transplant (1-year); **(b)** Patient survival across the full follow-up (0–15 years). Shaded bands indicate 95% confidence intervals; numbers below the x-axis show patients at risk at each time point. Log-rank p-values.

In sex-stratified Kaplan–Meier analyses (**Figures 1A, B**), no statistically significant differences in patient survival were observed between males and females. For 1-year patient survival (**Figure 1A**), the estimated probability at 1 year was 92.06% (95% CI 87.81–94.87) in males and 96.71% (95% CI 92.28–98.62) in female, again with no statistically significant difference (log-rank p=0.082). For patient survival (**Figure 1B**), the estimated survival probabilities at 5, 10 and 15 years were 73.38% (95% CI 66.1–79.3.8), 57.1% (95% CI 47.29–65.8), 36.01% (95% CI 22.4-49.7), in males, and 80.7% (95% CI 72.0–87.0), 72.9% (95% CI 62.1–81.8), 70.3 (95% CI 58.5-79.3) in females, the difference between curves was statistically significant (log-rank p=0.006). Era-stratified analyses showed that the estimated 1-year patient survival probabilities were 94.05% (95% CI, 86.29–97.48) for 2006–2011, 94.62% (95% CI, 89.53–97.27) for 2012–2018, and 92.95% (95% CI, 86.05–97.58) for 2019–2025. Although survival estimates appeared slightly higher after 2012, the differences were not statistically significant (log-rank p = 0.859).

**Figure 2:**
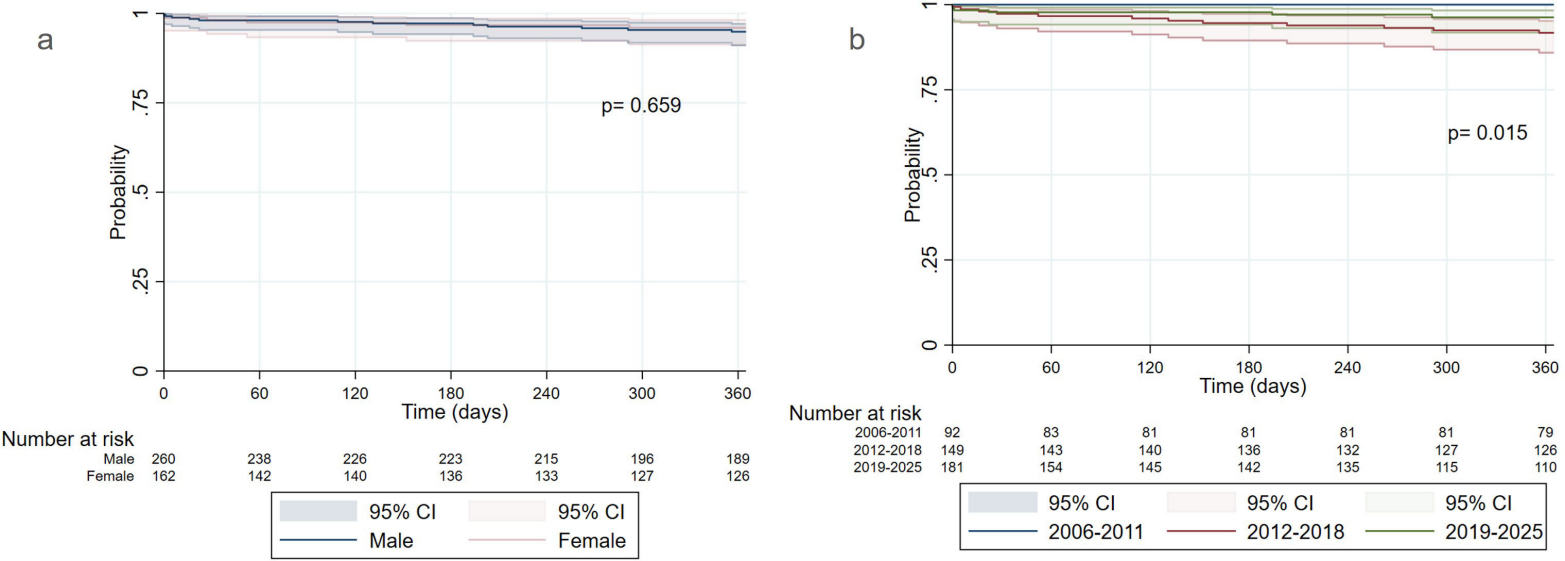
Graft survival within the first year by sex and era. 1-year graft survival by sex **(A)** and by transplant era **(B)**. Kaplan–Meier curves show the probability of graft survival during the first 1-year post-transplant; shaded bands indicate 95% confidence intervals, and numbers at risk are provided below each panel. **(A)** Male vs. female recipients (log-rank p). **(B)** Eras defined as 2006–2011, 2012–2018, and 2019–2025 (log-rank p).

Graft survival at 1 year was high and comparable between sexes (**Figure 2A**). Among males, the estimated Graft survival probability was 94.87% (95% CI: 91.12–97.06), while among females it was 95.98% (95% CI: 91.26–98.18). The Kaplan–Meier curves showed similar trajectories for both groups, with no statistically significant difference according to the log-rank test (p = 0.659). As shown in **Figure 2B**, graft survival at 1 year varied significantly across transplant eras (log-rank p = 0.015). The graft survival probability for the 2006–2011 era was 100%, for the 2012–2018 era, the estimated 1 year graft survival was 91.72% (95% CI: 85.87–95.21), while in the 2019–2025 era, survival improved to 96.26% (95% CI: 91.78–98.32).

**Figure 3:**
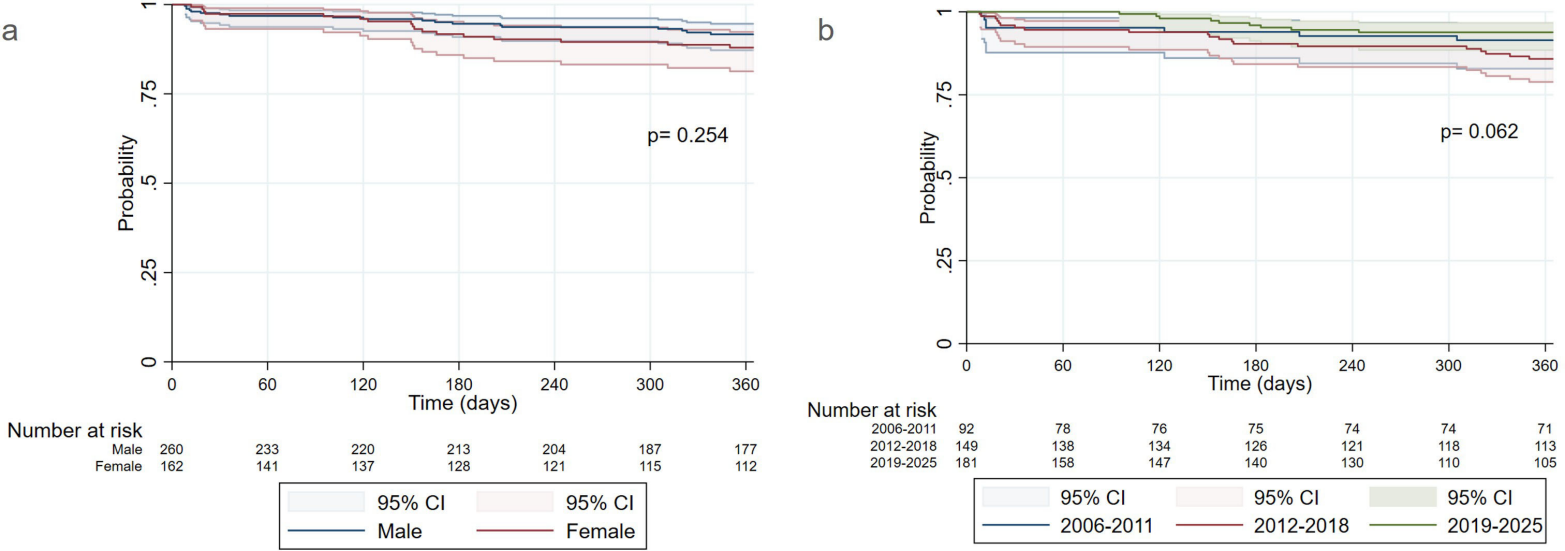
Graft rejection -free within the first year by sex and era. Kaplan–Meier curves showing the probability of graft rejection during the first year after kidney transplantation, stratified by sex **(a)** and transplant era **(b)**. Shaded areas represent 95% confidence intervals, and the number of patients at risk is shown below each plot. **(a)** Comparison between male and female recipients (log-rank p). **(b)** Comparison among transplant eras 2006–2011, 2012–2018, and 2019–2025 (log-rank p).

As shown in **Figure 3A**, rejection-free graft survival at 1 year did not differ significantly between sexes (log-rank p = 0.254). Among males, the estimated probability of remaining free from rejection at 1-year was 91.67% (95% CI: 87.2–94.6), in females, it was 87.95% (95% CI: 81.3–92.3). As shown in **Figure 3B**, statistically no significant differences significant were observed in 1-year rejection-free survival across transplant eras (log-rank p = 0.062). In the 2006–2011 group, the estimated probability was 91.44% (95% CI: 82.87–95.83). For the 2012–2018 era, the probability was 85.82% (95% CI: 78.86–90.62). Conversely, in the 2019–2025 group, rejection-free survival improved, reaching 93.81% (95% CI: 88.44–96.73).

**Figure 4:**
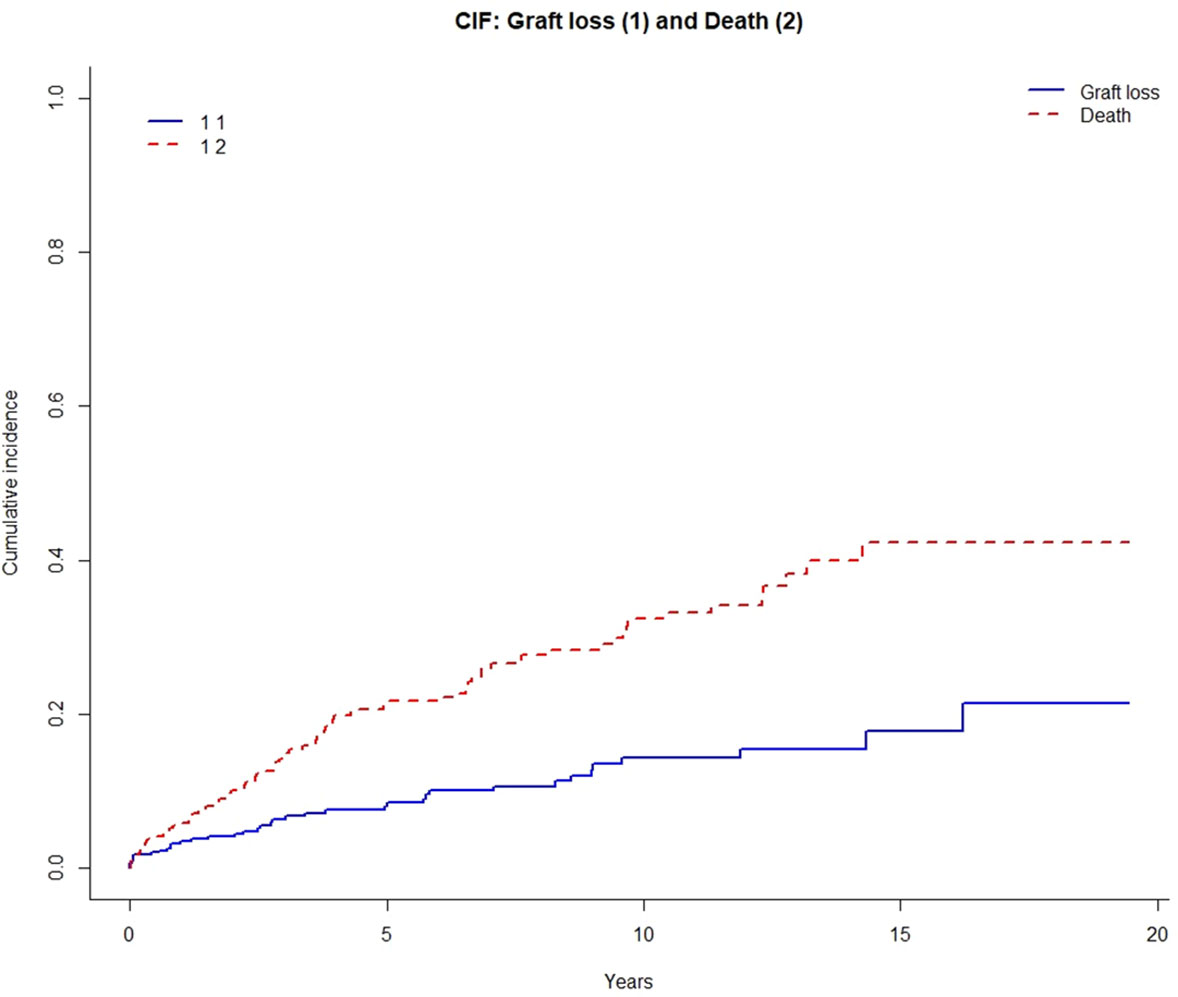
Competing risks over 15 years post–kidney transplant, cumulative incidence of death and graft loss.

In the 15-year cumulative incidence analysis, death occurred more frequently than graft loss. At 5 years, the cumulative incidence of graft loss was 8.0%, whereas death reached 21.6%. At 10 years, incidences were 14.3% for graft loss and 32.3% for death. By 15 years, the cumulative incidence of graft loss was 17.8%, compared with 42.2% for death (**Figure 4**).

## 4 Discussion

In this single-center Colombian cohort, overall and graft survival were favorable and broadly consistent with contemporary international reports. When stratified by sex, we did not detect statistically significant differences in either overall or 1-year graft survival, consistent with studies showing that crude survival differences between women and men are generally small. Observed disparities in some settings likely reflect modifiable clinical factors and donor–recipient matching (immunologic risk, size/sex mismatch) rather than intrinsic sex effects. Recent studies likewise indicate that post-transplant survival benefits are comparable between sexes, with no clear disadvantage for women (18) (19).

In our cohort, men represented the majority of recipients and were significantly older than women. This pattern is consistent with global transplant registries reporting a younger age and lower anthropometric measures among female recipients (20) (21). Diabetes mellitus was more prevalent among male recipients, whereas systemic lupus erythematosus, a recognized cause of CKD in younger women, was markedly more frequent in females (22). The most common etiology of CKD was hypertensive nephroangiosclerosis, slightly more frequent in women, reflecting the high burden of vascular disease in this population (23). Prior smoking was significantly more common among men, which may partially explain their higher prevalence of vascular comorbidity and cardiovascular risk (24).

Temporal analyses revealed a significant improvement in 1-year graft survival and a non-significant trend toward better rejection-free survival in the most recent era. These findings likely reflect advances in induction and maintenance immunosuppressive protocols, closer therapeutic drug monitoring, and greater adherence to standardized diagnostic frameworks for rejection (25). In addition, transplant practices at our institution evolved over the study period, including refinements in surgical techniques, improvements in perioperative care, updates in immunosuppressive strategies, and more standardized post-transplant follow-up, which may have contributed to the progressive improvement in outcomes observed across different eras. It should be noted that the most recent period (2019–2025) partially overlapped with the COVID-19 pandemic, which temporarily affected transplant activity and healthcare delivery worldwide. During this period, many transplant programs experienced reduced surgical volume, delays in follow-up, and modifications in clinical management. These circumstances may have influenced outcomes in this era and should be considered when interpreting temporal comparisons. We also observed an increase in living donation among women in the most recent period (26).

A salient feature of our long-term follow-up is that mortality exceeded graft loss as the predominant competing event by 15 years. This pattern mirrors international data in which cardiovascular disease, infections, and malignancy increasingly drive late mortality despite functioning allografts (27). At 15 years, cumulative mortality reached 42.2%, while graft loss was 17.8%, emphasizing the need for longitudinal care focused on extra-renal outcomes. Regionally, Colombian cohorts using competing-risk methods have also shown substantial cumulative incidence of death alongside graft loss, and have highlighted risk factors such as BK polyomavirus nephropathy (BKPyVAN), acute rejection, and CMV disease (17). Our cumulative incidence curves therefore situate the program within both global and Latin-American experience, and underscore the need to manage competing risks proactively through rigorous cardiovascular prevention, infection surveillance, and cancer screening. Programmatic continuity of care may be critical: a recent national administrative-data study linked higher health-care fragmentation during the first post-transplant year with worse 3-year survival (28).

Additionally, the predominance of hypertensive nephroangiosclerosis as the leading cause of ESRD in our cohort contrasts with reports from the United States, where diabetes mellitus predominates (7). This difference may reflect variations in population characteristics, access to early diagnosis and management of diabetes, as well as potential differences in diagnostic classification in routine clinical practice. Due to the low proportion of living donor transplants in our cohort, we did not perform formal comparative analyses between living and deceased donor recipients, as the study was underpowered to detect meaningful differences in outcomes.

The relatively low proportion of living donor transplantation in our cohort may reflect structural and cultural factors, including reliance on deceased-donor allocation systems, limited access to living donor programs, and differences in societal attitudes toward donation, in contrast to countries such as Japan, where living donation predominates (29).

### 4.1 Limitations

As a retrospective single-center analysis, results may not be generalizable to other institutions with different patient profiles, surgical expertise, or resource availability. Second, although we applied robust data capture through institutional registries and REDCap validation, the possibility of residual misclassification cannot be excluded. Third, although temporal comparisons suggest improvements in outcomes across eras, these findings may be influenced by unmeasured confounders, including secular changes in donor selection, immunosuppressive strategies, and supportive care. In addition, the duration of follow-up was limited, with a relatively small proportion of patients reaching extended follow-up periods, which may reduce the precision of long-term survival estimates. Additionally, post-transplant renal function was only available at hospital discharge, which limited the ability to assess early functional recovery. Finally, causes of mortality (coronary disease, malignancy, infections) and graft failure (rejection, polyomavirus infection, vascular injury) were not described, as this information was not available, which should be considered when interpreting the results.

## 5 Conclusions

In this 19-year institutional experience, kidney transplantation achieved patient and graft survival outcomes that match international benchmarks, underscoring high-quality performance in a middle-income Latin American setting. We observed era-to-era improvements in graft survival and overall care standardization, with broadly equitable outcomes across sexes. The main bottleneck now lies beyond perioperative care: late mortality, rather than graft failure, has emerged as the main long-term challenge. This finding suggests that long-term outcomes are increasingly driven by non-graft-related factors, particularly cardiovascular disease, infections, and malignancy, highlighting the need for comprehensive post-transplant care beyond graft preservation.

## Data Availability

The original contributions presented in the study are included in the article/supplementary material. Further inquiries can be directed to the corresponding author.
